# Positive allosteric modulation of a GPCR ternary complex

**DOI:** 10.1126/sciadv.adp7040

**Published:** 2024-09-11

**Authors:** Wessel A. C. Burger, Christopher J. Draper-Joyce, Celine Valant, Arthur Christopoulos, David M. Thal

**Affiliations:** ^1^ Drug Discovery Biology, Monash Institute of Pharmaceutical Sciences, Monash University, Parkville, Victoria 3052, Australia.; ^2^ Australian Research Council Centre for Cryo-Electron Microscopy of Membrane Proteins, Monash Institute of Pharmaceutical Sciences, Monash University, Parkville, Victoria 3052, Australia.

## Abstract

The activation of a G protein–coupled receptor (GPCR) leads to the formation of a ternary complex between agonist, receptor, and G protein that is characterized by high-affinity binding. Allosteric modulators bind to a distinct binding site from the orthosteric agonist and can modulate both the affinity and the efficacy of orthosteric agonists. The influence allosteric modulators have on the high-affinity active state of the GPCR-G protein ternary complex is unknown due to limitations on attempting to characterize this interaction in recombinant whole cell or membrane-based assays. Here, we use the purified M_2_ muscarinic acetylcholine receptor reconstituted into nanodiscs to show that, once the agonist-bound high-affinity state is promoted by the G protein, positive allosteric modulators stabilize the ternary complex that, in the presence of nucleotides, leads to an enhanced initial rate of signaling. Our results enhance our understanding of how allosteric modulators influence orthosteric ligand signaling and will aid the design of allosteric therapeutics.

## Teaser

G proteins and positive allosteric modulators enhance GPCR signaling.

## INTRODUCTION

G protein–coupled receptors (GPCRs) are cell surface proteins activated by extracellular environmental stimuli and, in response, facilitate the modulation of intracellular signaling pathways and biological responses (*1*). GPCRs are important drug targets, and understanding how different ligands and heterotrimeric G proteins interact and modulate GPCR activity can facilitate the design of future therapeutics (*2*). A milestone in GPCR pharmacology was the observation that agonists have low- and high-affinity binding states for a receptor that was dependent on the presence of guanine nucleotides (*3*). The low-affinity state (*K*_Low_) arises from the binding between an agonist and receptor, whereas the high-affinity state (*K*_High_) represents a ternary complex between an agonist, receptor, and G protein, with the increase in binding affinity being due to positive cooperativity between the agonist and G protein (*4*, *5*). These observations and others (*6*) form the basis of the ternary complex model (TCM) (*7*), a central paradigm in modern GPCR pharmacology that posits transducer proteins such as G proteins are endogenous positive allosteric modulators (PAMs) of agonist-GPCR complexes.

Structural biology studies have provided mechanistic insight into the interactions of agonists and G proteins with receptors (*8*–*10*). The binding of agonists to GPCRs promotes conformational rearrangements within GPCRs that activate the receptor and accommodate the intracellular binding of heterotrimeric G proteins and other transducer proteins (*10*). Nuclear magnetic resonance (NMR) experiments revealed that agonist binding alone does not fully stabilize the active receptor conformation and requires interaction with the G protein (*11*–*13*), supporting the TCM. Further interrogation of high-affinity agonist binding was made possible by reconstituting purified GPCRs into nanodiscs (*14*, *15*). Nanodiscs provide a stable platform to interrogate the relative ratios of a GPCR to a G protein in a reductionist approach that allows the determination of the low-affinity (*K*_Low_) and high-affinity (*K*_High_) states away from the cellular milieu. Consistent with the TCM was the observation that shifts in agonist affinity (*K*_Low_/*K*_High_) promoted by transducers correlated strongly with molecular efficacy, suggesting that the transducer cooperativity can be used as a surrogate measure of efficacy (*16*). Pharmacological experiments using β_2_AR nanodiscs showed that G proteins could alter the association and dissociation rates of orthosteric ligands, which implies an allosteric coupling between agonist and transducer binding, further validating the TCM (*17*).

**Fig. 1.**
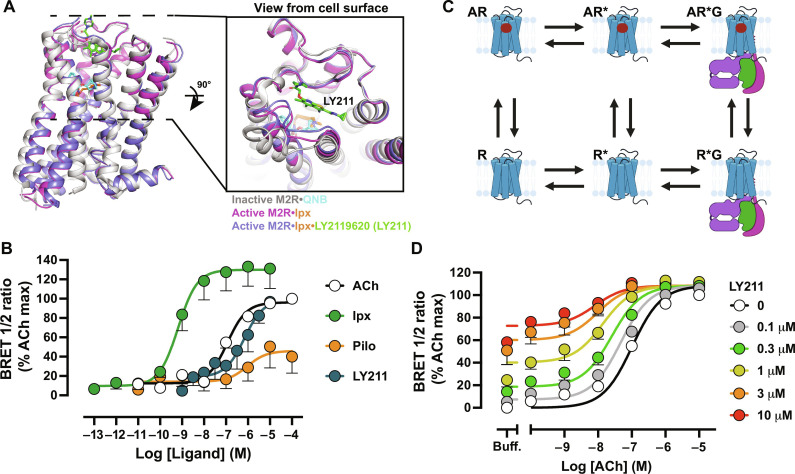
Allosteric receptor and G protein activation at the M_2_ mAChR. (**A**) Comparison of the inactive M_2_ mAChR structure bound to QNB [Protein Data Bank (PDB): 3UON; receptor colored white and QNB in cyan] to the active M_2_ mAChR structure bound to Ipx (PDB: 4MQS; receptor colored magenta with Ipx in orange) and to the active + PAM M_2_ mAChR structure bound to Ipx and LY211 (PDB: 4MQT; receptor colored blue with Ipx in orange and LY211 in green). (**B**) G_i1_ activation in M_2_ mAChR FlpIn CHO cells. Concentration-response curves of ACh, Ipx, Pilo, and LY211. Data represent ligand-induced BRET normalized to the maximum response of Ipx. A three-parameter logistic equation was globally fit to the data. (**C**) Extended ternary complex where A is the agonist, G is the G protein, R is an inactive receptor, and R* is the active receptor. (**D**) G_i1_ activation in M_2_ mAChR FlpIn CHO cells. Interaction of LY211 and ACh. Data represent ligand-induced BRET normalized to the maximum response of ACh. The operational model of allosterism was globally fit to the data. Data represent the means ± SEM of at least three individual experiments performed in duplicate. Group sizes and obtained parameters are listed in Table 1. Figure 1C was created with BioRender.com.

Most GPCRs have secondary, spatially distinct binding sites known as allosteric sites (*9*). In recent years, there has been increased interest in the discovery and development of small-molecule allosteric modulators that target allosteric sites because these binding sites offer the potential for the design of more selective ligands in comparison to orthosteric ligands that target conserved orthosteric sites (*18*–*20*). An exemplar system to study GPCR allostery is the muscarinic acetylcholine receptors (M_1_ to M_5_ mAChRs) (*21*–*23*). The first PAM-bound structure was the M_2_ mAChR in complex with a G protein mimetic, the agonist iperoxo (Ipx), and the PAM LY2119620 (LY211; Fig. 1A). The structure revealed that LY211 bound in a large allosteric binding site located above the orthosteric site in a solvent-accessible extracellular vestibule (ECV) that was in a closed conformation (*24*). In another study, bitopic probes having an orthosteric agonist component and a component spatially restricted to the ECV showed that the conformation of the ECV influenced the signaling capacity of the M_2_ mAChR. Similarly, recent mutagenesis studies showed that ACh affinity was enhanced when residues in the ECV were mutated (*25*, *26*). Together, these studies highlight the allosteric coupling between allosteric and orthosteric binding sites.

The combined effect of G proteins (endogenous PAMs) and exogenous small-molecule PAMs on orthosteric agonist affinity and how this influences signaling are poorly established. In this study, we investigated how the PAM LY211 and G proteins influence agonist binding and signaling at GPCRs by reconstituting purified human M_2_ mAChRs into nanodiscs. The degree of cooperativity from the low-affinity to the high-affinity state promoted by the G protein was dependent on the efficacy of the orthosteric agonist and was not increased further by adding a PAM. This indicates that, once the high-affinity state is promoted by the G protein, the PAM cannot further increase orthosteric agonist affinity. Investigation of the kinetics of the allosteric interaction between a PAM and the ternary complex revealed a decrease in the association and dissociation rates of the orthosteric ligand from the ternary complex in the presence of the PAM. By measuring the activation kinetics of G protein in the presence of allosteric modulators, we show that the PAM more readily stabilizes the ternary complex, leading to an enhanced rate of signaling by the G protein. These results further our understanding of how small-molecule PAMs affect the high-affinity state and GPCR signaling.

## RESULTS

### G protein activation at the M_2_ mAChR

Following agonist binding, the agonist-bound receptor binds to and activates a G protein heterotrimer, leading to the dissociation of the Gα and Gβγ subunits (*27*). Wild-type (WT) M_2_ mAChR expressing FlpIn Chinese hamster ovary (CHO) cells were transiently transfected with the TRUPATH bioluminescence resonance energy transfer (BRET) Gα_i1_β_3_γ_9_ sensors (*28*), and the decrease in BRET signal representative of subunit dissociation (and therefore G protein activation) in response to different agonists was measured. The orthosteric agonists ACh, Ipx, pilocarpine (Pilo), and the agonist-PAM (Ago-PAM) LY211 promoted different extents of receptor-mediated G protein activation and efficacy (Fig. 1B and Table 1). As per the extended TCM (ETCM), which builds on the TCM to account for receptor isomerization from the inactive to the active state (R to R*) before engagement of the R* state with G protein (R*G) (*29*) (Fig. 1C), this suggests that orthosteric and allosteric agonists differ in their ability to promote the conversion of AR to AR*. We considered ACh the reference full agonist because it is the endogenous agonist. Ipx produced a maximal response greater than ACh and displayed greater efficacy (Table 1). For these reasons, Ipx was considered a “super agonist” in this system, consistent with prior studies at the M_2_ mAChR (*30*, *31*), indicating that Ipx has a greater capacity to form the AR* state than ACh. Conversely, Pilo is a partial agonist, as evidenced by its decreased efficacy relative to ACh (Table 1), and weakly promotes the conversion of the AR to AR* state. In the case of the Ago-PAM LY211, a saturating effect of G protein activation was not reached as solubility issues of the compound prevented the use of higher concentrations. As such, the reported maximal effect and efficacy values represent estimates. However, LY211 appeared to promote a similar maximal response as ACh. This illustrates that receptor activation can occur due to agonist binding at either the orthosteric or an allosteric site. In addition, a concentration-response curve of ACh with increasing concentrations of LY211 revealed that ACh signaling was potentiated in the presence of LY211 (Fig. 1D). The data were analyzed with an operational model of allosterism to derive a functional cooperativity factor (αβ) (*32*). The positive log αβ value indicates that LY211 enhances the ability of ACh to promote receptor-mediated G protein activation (conversion from R state to R* state) (Fig. 1D and Table 1). It is difficult to characterize the R*G state in functional assays due to the transient nature of this state in the presence of nucleotides; as such, we measured high-affinity binding (occurring at the R*G state) in radioligand binding experiments using M_2_ mAChR reconstituted into nanodiscs with purified WT G protein heterotrimer (Gα_i1_β_1_γ_2_).

**Table 1.** Pharmacological parameters from functional and radioligand binding experiments. Data represent the means ± SEM of (*x*) independent experiments performed in duplicate. N.D., not determined; N.A., not applicable.

Functional parameters of ligands in a G_i1_ (TRUPATH) protein activation assay in M_2_ mAChR CHO cells										
Parameter			ACh	Ipx		Pilo		LY211		
pEC_50_^*^			6.94 ± 0.11 (7)	9.18 ± 0.22 (7)		6.03 ± 0.78 (6)		6.17 ± 0.29 (4)		
Log τ^†^			1.23 ± 0.08 (7)	1.61 ± 0.25 (7)		0.13 ± 0.16 (6)		0.75 ± 0.12 (4)		
(LY211) - Log αβ^‡^			1.72 ± 0.19 (3)	N.D.		N.D.		N.A.		
p*K*_B_^§^			N.A.	N.A.		N.A.		5.43 ± 0.15 (3)		
**[^3^H]-NMS competition binding assays at M_2_ mAChR nanodiscs with G_i1_ protein or M_2_ mAChR CHO membrane**										
**Frac_Hi_ - M_2_ mAChR nanodiscs - receptor:G protein molar ratio^¶^**									**M_2_ mAChR membrane^¶^**	
	**1:0**	**1:10**	**1:50**	**1:100**	**1:200**	**1:500**	**1:1000**	**1:2000**	**+ Gpp[NH]pp**	**− Gpp[NH]pp**
ACh	0.31 ± 0.08 (7)	0.61 ± 0.08 (4)	0.61 ± 0.08 (4)	0.78 ± 0.08 (3)	0.76 ± 0.09 (4)	0.75 ± 0.08 (6)	0.83 ± 0.08 (6)	1.00 ± 0.10 (6)	0.13 ± 0.14 (3)	0.53 ± 0.11 (3)
Ipx	0.12 ± 0.04 (11)	N.D.	N.D.	N.D.	N.D.	0.68 ± 0.06 (3)	0.76 ± 0.06 (3)	0.91 ± 0.05 (3)	N.D.	N.D.
Pilo	0.01 ± 0.06 (11)	N.D.	N.D.	N.D.	N.D.	0.50 ± 0.10 (3)	0.55 ± 0.10 (3)	0.73 ± 0.10 (3)	N.D.	N.D.
**Low and high affinity**										
				**p*K*_i(Low)_^#^**	**p*K*_i(High)_^#^**		(G protein) - Log α^**^	**p*K*_i_ + 10 μM LY211**		**p*K*_i_ + 10 μM LY211 + G_i1_**
ACh		Nanodisc		5.72 ± 0.13 (7)	6.98 ± 0.09 (7)^††^		1.28 ± 0.12 (7)	7.13 ± 0.10 (7)^††,‡‡^		7.18 ± 0.15 (4)^††,‡‡^
		Membrane		5.84 ± 0.12 (3)	7.25 ± 0.69 (3)		N.D.	N.D.		N.D.
Ipx		Nanodisc		7.72 ± 0.06 (11)	9.77 ± 0.10 (4)^††^		2.33 ± 0.18 (3)	9.77 ± 0.18 (3)^††,§§^		9.84 ± 0.10 (3)^††,§§^
Pilo		Nanodisc		5.33 ± 0.06 (11)	6.43 ± 0.17 (4)^††,¶¶^		0.97 ± 0.10 (3)	5.38 ± 0.12 (8)^§§^		6.02 ± 0.18 (4)^††,§§,¶¶^
**[^3^H]-NMS interaction binding assays with LY211 and/or G protein at M_2_ mAChR nanodiscs**										
**Full interaction**										
					**(LY211) - p*K*_B_^##^**	(LY211) - Log α_Agonist_^***^			**(LY211) - Log α_[3H]-NMS_^†††^**	
− G protein heterotrimer		ACh			6.09 ± 0.15 (15)	0.65 ± 0.13 (7)			−0.29 ± 0.04 (15)	
		Ipx				2.05 ± 0.21 (4)				
		Pilo				0.12 ± 0.14 (4)				
+ 1:2000 G protein heterotrimer		ACh			6.92 ± 0.16 (4)	= 0^‡‡‡^			−0.37 ± 0.04 (4)	
**Titration**										
			**p*K*_B_^##^**				**Log α_[3H]-NMS_^†††^**			
LY2119620			5.84 ± 0.60 (3)				−0.19 ± 0.08 (3)			
G protein heterotrimer			7.37 ± 0.22 (10)				−0.55 ± 0.16 (10)			
LY2119620 + 1:2000 G protein heterotrimer			6.62 ± 0.27 (3)				−0.81 ± 0.20 (3)			
**[^3^H]-NMS association and dissociation assays at M_2_ mAChR nanodiscs**										
	**[^3^H]-NMS**		**+10 μM LY211**			**+ G_i1_**		**+10 μM LY211 + G_i1_**		
*k*_off_^§§§^	0.150 ± 0.011 (4)		0.049 ± 0.005 (4)^¶¶¶^			0.097 ± 0.017 (3)^¶¶¶^		0.031 ± 0.013 (3)^¶¶¶,###^		
*k*_on_^****^	5.50 ± 0.66 × 10^8^ (5)		2.35 ± 0.43 × 10^8^ (3)^¶¶¶^			2.52 ± 0.29 × 10^8^ (3)^¶¶¶^		2.30 ± 0.39 × 10^8^ (3)^¶¶¶^		
*B*_max_^††††^	131 ± 4.71 (3)		115 ± 13.2 (3)			114 ± 11.4 (3)		20.33 ± 4.96 (3)^‡‡‡‡^		
**Kinetic Gα_i1_ (TRUPATH) protein activation assay in M_2_ mAChR CHO cells**										
	**ACh**		**Ipx**		**LY211**		**ACh + LY211**		**Ipx + LY211**	
Initial rate^§§§§^	290 ± 9.79 (10)		217 ± 6.48 (3)		198 ± 51.0 (3)		564 ± 30.6 (4)		586 ± 26.8 (4)	
Steady state^¶¶¶¶^	103 ± 0.84 (10)		102 ± 0.95 (3)		69.1 ± 4.22 (3)		123 ± 1.00 (4)^####^		114 ± 0.68 (4)^####^	

*Negative logarithm of the concentration of agonist required to give a half-maximal response obtained from the three-parameter logistic equation.

†Logarithm of operational efficacy parameter obtained from the operational model of agonism.

‡Logarithm of the αβ cooperativity factor for the interaction between LY211 and ACh obtained from the operational model of allosterism.

§Negative logarithm of the allosteric modulator equilibrium dissociation constant (p*K*_B_) obtained from the operational model of allosterism.

¶Fraction of receptors found in the high-affinity state obtained from the two-site competition binding model.

#Negative logarithm of the low [p*K*_i(Low)_] or high [p*K*_i(High)_] orthosteric equilibrium dissociation constant obtained from the two-site competition binding model.

**Logarithm of the α cooperativity factor for the interaction between an agonist and G protein obtained from the ATCM.

††Significantly different from p*K*_i(Low)_ as determined by one-way ANOVA, Tukey’s multiple comparisons, *P* < 0.05.

‡‡Negative logarithm of the orthosteric equilibrium dissociation constant at a PAM-occupied receptor (p*K*_I_ + log α_ACh_) obtained from the ATCM.

§§Negative logarithm of the orthosteric equilibrium dissociation constant (p*K*_i_) obtained from the one-site competition binding model.

¶¶Significantly different from p*K*_i_ Pilo + 10 μM LY211 as determined by one-way ANOVA, Tukey’s multiple comparisons, *P* < 0.05.

##Negative logarithm of the allosteric modulator equilibrium dissociation constant (p*K*_B_) obtained from the ATCM.

***Logarithm of the α cooperativity factor for the interaction between an orthosteric agonist and LY211 obtained from the ATCM.

†††Logarithm of the α cooperativity factor for the interaction between [^3^H]-NMS and LY211 obtained from the ATCM.

‡‡‡Value was not statistically different to zero as determined by *F* test and was therefore constrained to zero.

§§§Dissociation rate of [^3^H]-NMS in min^−1^ obtained from the monoexponential decay model.

¶¶¶Significantly different from [^3^H]-NMS only as determined by one-way ANOVA, Tukey’s multiple comparisons, *P* < 0.05.

###Significantly different from [^3^H]-NMS + G_i1_ only as determined by one-way ANOVA, Tukey’s multiple comparisons, *P* < 0.05.

****Association rate of [^3^H]-NMS in M^−1^ s^−1^ with *k*_off_ constrained to the value given for §§§ obtained from the one-phase association model.

††††Maximal binding at equilibrium, extrapolated to maximal radioligand concentration obtained from the one-phase association model.

‡‡‡‡Significantly different from [^3^H]-NMS only, [^3^H]-NMS + 10 μM LY211, and [^3^H]-NMS + G_i1_ as determined by one-way ANOVA, Tukey’s multiple comparisons, *P* < 0.05.

§§§§Initial rate of signaling for G protein activation in the presence of a saturating amount of drug in BRET units min^−1^ baseline then rise to steady state with drift model.

¶¶¶¶Final effect level stimulated by a saturating amount of drug baseline then rise to steady state with drift model.

####Significantly different from ACh/Ipx only and LY211 only as determined by one-way ANOVA, Šidák’s multiple comparisons, *P* < 0.05.

### Reconstitution of the M_2_ mAChR into nanodiscs

To generate M_2_ mAChR nanodiscs, we used a modified receptor construct (M_2ΔICL3_ mAChR) that was also used in the x-ray crystallography and cryo–electron microscopy studies of the M_2_ mAChR. Expression and purification of M_2ΔICL3_ mAChR were performed as previously described (fig. S1, A to C) (*24*, *33*). To verify that this receptor construct was functional, we stably transfected the M_2ΔICL3_ mAChR construct into CHO cells. Radioligand binding data indicated that the subsequent cell line was low expressing. Instead, we turned to a highly amplified functional assay and determined extracellular signal–regulated kinase 1/2 phosphorylation (pERK1/2) in response to ACh and Ipx (fig. S1D). As expected for a low-expressing cell line, the obtained pEC_50_ values for ACh and Ipx resemble reported affinity values at the M_2_ mAChR. Furthermore, the rank order of agonism and potency, as well as the fold difference in potency between ACh and Ipx, was maintained between WT and M_2ΔICL3_ mAChR, validating the functionality of this receptor construct (*24*). Purified M_2ΔICL3_ mAChR was reconstituted into nanodiscs using a covalently circularized membrane scaffold protein (MSP), cMSP1D1 (fig. S2, A and B) (*34*), and the monomeric nature of the nanodisc was verified through complexing with an anti-Flag Fab and negative stain electron microscopy (fig. S2, C to E). Hereafter, we refer to reconstituted M_2ΔICL3_ mAChR in nanodiscs as M_2_ mAChR nanodiscs.

### Formation and characterization of the high-affinity state at the M_2_ mAChR

To characterize the high-affinity state of M_2_ mAChR nanodiscs, we first expressed and purified a range of heterotrimeric G proteins with differing Gα subunits and Gβ_1_γ_2_ (fig. S3, A and B). At a 1:2000 molar ratio of M_2_ mAChR:G protein (R:G), G_i1_, G_i2_, G_i3_, G_oA_, and G_s_ heterotrimers were able to promote a similar increase in the affinity of ACh in radioligand competition binding experiments with [^3^H]-*N*-methylscopolamine (NMS) (fig. S3C). G_q_ could not mediate any change in ACh affinity (fig. S3C). This G protein coupling profile was consistent with our recent findings of G protein signaling at the M_2_ mAChR using the TRUPATH assay (*35*). The observation that the noncanonical G_s_ promoted a similar shift in affinity at the M_2_ mAChR compared to the canonical G_i/o_ may be explained through the findings of a recent study where nucleotide-free G proteins display altered selectivity profiles (*36*).

**Fig. 2.**
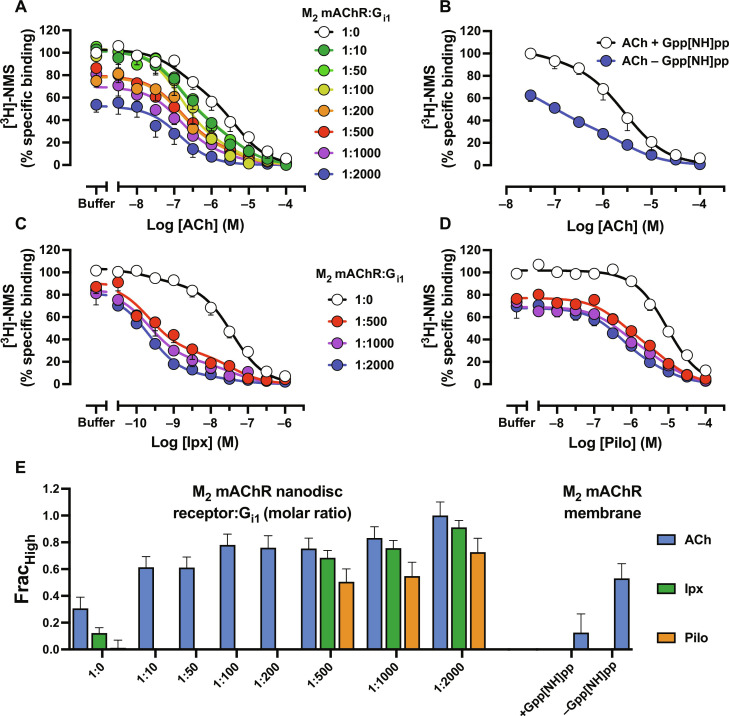
Modulation of the low- and high-affinity state of orthosteric agonists by G protein. (**A**) Radioligand competition binding between ACh and [^3^H]-NMS at M_2_ mAChR nanodiscs in the presence of stoichiometric amounts of receptor to G_i1_ protein. (**B**) Radioligand competition binding between ACh and [^3^H]-NMS at M_2_ mAChR membranes with and without 100 μM Gpp[NH]pp. (**C**) Radioligand competition binding between Ipx and [^3^H]-NMS at M_2_ mAChR nanodiscs in the presence of stoichiometric amounts of receptor to G_i1_ protein. (**D**) Radioligand competition binding between Pilo and [^3^H]-NMS at M_2_ mAChR nanodiscs in the presence of stoichiometric amounts of receptor to G_i1_ protein. Data were normalized to the buffer-only condition. A two-state model of competition binding with p*K*_i(Low)_ and p*K*_i(High)_ shared was globally fit to the data. For all panels, data represent the means ± SEM of three individual experiments performed in duplicate. Group sizes and obtained parameters are listed in Table 1. (**E**) Fraction of M_2_ mAChR receptors found in the high-affinity state in the presence of different ligands, G protein ratios, or Gpp[NH]pp from (A) to (D).

Given the lack of difference between canonical G_i/o_ proteins in increasing ACh affinity, we performed a complete characterization of the high-affinity state with one of these G proteins, specifically G_i1_. Titrating amounts of G_i1_ heterotrimer at M_2_ mAChR nanodiscs increased the fraction of receptors in the high-affinity state (Frac_Hi_ values; Fig. 2A). At the 1:2000 R:G ratio, all of the receptor population was in the high-affinity state, indicating that this R:G ratio was sufficient for complete formation of the high-affinity state (Fig. 2E). Formation of the active state was accompanied by a loss of [^3^H]-NMS binding, consistent with destabilization of the inactive conformation of the receptor. Analyzing the low- and high-affinity states of ACh with a two-site competition binding model with p*K*_i(Low)_ and p*K*_i(High)_ values shared across the R:G ratios revealed an 18-fold increase in the affinity of ACh between p*K*_i(Low)_ and p*K*_i(High)_ (Table 1). To verify that an incubation period of 6 hours at room temperature (RT) was sufficient for nucleotide-free G protein to bind M_2_ mAChR nanodiscs and reach equilibrium, we determined p*K*_i(Low)_ and p*K*_i(High)_ values following a shorter incubation period. Similar p*K*_i(Low)_ and p*K*_i(High)_ values were obtained at 4 hours, indicating that equilibrium was already reached before 6 hours (fig. S4A) (*37*). To ensure receptor stability, we measured [^3^H]-NMS binding and determined nanodisc integrity through size exclusion chromatography (SEC) over a 24-hour period at RT (fig. S4, B and C). Both approaches validated nanodisc stability and integrity for up to 6 hours, with the 24-hour time point leading to a 37% loss in [^3^H]-NMS binding.

To compare ACh affinity values at M_2_ mAChR nanodiscs to those observed in a more native cell environment, we performed competition binding between ACh and [^3^H]-NMS at M_2_ mAChR FlpIn CHO membranes (Fig. 2B). During membrane preparation, endogenous nucleotide is lost and must be added exogenously in the form of a GTP analog, 5′-guanylyl imidodiphosphate (Gpp[NH]pp). By performing competition binding with and without Gpp[NH]pp, ACh affinity at the receptor alone [p*K*_i(Low)_] and at the ternary complex [p*K*_i(High)_] can be determined (*4*, *38*). In the absence of Gpp[NH]pp, around half of the receptors were in the high-affinity state (Fig. 2E), highlighting the difficulty in fully forming the high-affinity state in membranes compared to nanodiscs. It is possible that there was an insufficient concentration of G protein within these membrane preparations to fully form the active state. Alternatively, the presence of asymmetric dimers in membrane preparations, where only one receptor subunit is coupled to a G protein and in the high-affinity state, could explain the lower fraction of receptors in the high-affinity state in membranes (*39*, *40*). Despite this, similar low- and high-affinity values were obtained with M_2_ mAChR membranes compared to M_2_ mAChR nanodiscs, justifying the use of nanodiscs to explore, in a more exclusive manner, the high-affinity state at the M_2_ mAChR (Table 1).

Given the difference in the ability of agonists to redistribute the R to R* equilibrium according to a G protein activation assay (Fig. 1B), we explored the impact of a super and partial agonist (Ipx and Pilo) on the high-affinity state. Competition binding between Ipx and Pilo versus [^3^H]-NMS was performed in the presence of the three highest stoichiometric amounts of G protein (Fig. 2, C to E). For Ipx, similar Frac_Hi_ values as to those observed with ACh were seen (Fig. 2, C to E), although there was a greater shift (112-fold) in Ipx affinity versus ACh (18-fold). In the presence of Pilo, a smaller fraction of receptors were in the high-affinity state compared to ACh, which is consistent with Pilo being a partial agonist (Fig. 2, D and E). In contrast, there was a smaller shift (13-fold) from the low- to high-affinity state with Pilo (Table 1).

Considering that G proteins act as an endogenous allosteric modulator of agonist binding, we calculated binding cooperativity values (α) that describe the change in orthosteric ligand affinity when co-bound with an allosteric modulator using the allosteric TCM (ATCM) (*19*, *41*). Positive values indicate positive cooperativity, negative values indicate negative cooperativity, and values of zero indicate neutral cooperativity. Consistent with our previously obtained p*K*_i(Low)_-p*K*_i(High)_ values, G_i1_ displayed a binding cooperativity rank order of Pilo < ACh < Ipx (Table 1). Similarly, the collapse in radioligand binding observed with increasing G protein concentrations indicates that the G protein acts as a negative allosteric modulator (NAM) of antagonist binding. Plotting the G protein ratios (G_i1_) as a molar concentration and using the ATCM revealed a binding cooperativity value of log α of −0.55 ± 0.16 for [^3^H]-NMS binding and a binding affinity between the G protein and the NMS-bound receptor of ~42 nM (fig. S6). Together, the differences between p*K*_i(Low),_ p*K*_i(High),_ Frac_Hi_, and α for agonists match the G protein activation assay results and validate that the degree of G protein activation and efficacy of orthosteric agonists exhibit the extent to which orthosteric agonists stabilize the active state. The difference in the direction of G protein cooperativity between agonists and antagonists reflects a two-state model of allostery and allows for the comparison of cooperativity between the endogenous G protein and exogenous allosteric modulators.

### Formation of the high-affinity state, G protein versus PAM

**Fig. 3.**
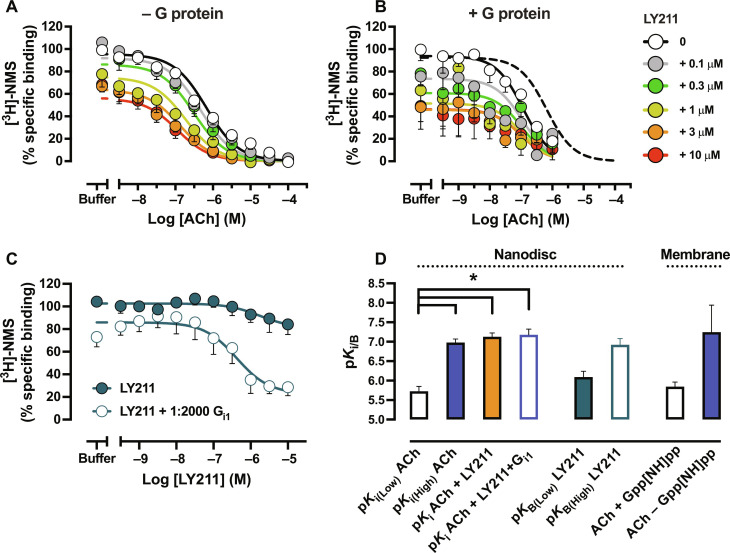
Modulation of the low- and high-affinity state of orthosteric ligands by G protein and PAM. Radioligand competition binding between ACh and [^3^H]-NMS at M_2_ mAChR nanodiscs with increasing amounts of LY2119620 (**A**) without and (**B**) with a saturating 1:2000 stoichiometric R:G ratio of G_i1_ protein. The shift in ACh affinity in the presence of G_i1_ is illustrated by including the ACh binding curve without G protein in (B) (dashed line). For both experiments, data were normalized to the buffer-only condition. An ATCM was globally fit to the data. (**C**) Allosteric interaction between LY211 and [^3^H]-NMS at M_2_ mAChR nanodiscs with and without a saturating 1:2000 stoichiometric R:G ratio of G_i1_ protein. Data were normalized to the buffer-only condition. An ATCM was globally fit to the data. (**D**) p*K*_i_ and p*K*_B_ values of ACh and LY211 were obtained in radioligand binding at M_2_ mAChR nanodiscs and M_2_ mAChR membranes. For all panels, data represent the means ± SEM of at least three individual experiments performed in duplicate. Group sizes and obtained parameters are listed in Table 1. *, significantly different, *P* < 0.05, one-way ANOVA, Tukey’s multiple comparisons test.

Similar to G proteins, PAMs increase orthosteric ligand affinity. However, how this relates to the ability of the G protein to form the high-affinity state remains unknown. To explore this, we initially tested the ability of LY211 to modulate ACh, Ipx, and Pilo affinity at M_2_ mAChR nanodiscs without the presence of G proteins in [^3^H]-NMS radioligand binding. Any increase in agonist affinity thus represents the ability of the PAM to modulate the low-affinity state of these agonists. Again, we used the ATCM (*19*, *41*) to derive binding cooperativity values. LY211 is a PAM of ACh, Ipx, and Pilo binding with a similar rank order compared to G protein, Pilo < ACh < Ipx (Fig. 3A, fig. S5, A and B, and Table 1). As evidenced by the collapse in [^3^H]-NMS binding, LY211 negatively modulates [^3^H]-NMS binding (log α_[3H]-NMS_ = −0.29 ± 0.04). The observed cooperativity values for LY211 are consistent with previous studies (Table 1) (*24*, *42*). Compared to LY211, our data indicate that G_i1_ is a superior PAM of agonist binding and a similar NAM of [^3^H]-NMS binding.

Next, we tested if LY211 could modulate the high-affinity state and focused on ACh by performing an interaction between ACh and LY211 in the presence of a saturating amount of G protein (R:G of 1:2000) (Fig. 3B). Increasing concentrations of LY211 produced no apparent change in the affinity of ACh, demonstrating that LY211 is a neutral allosteric ligand (log α_ACh_ = 0) of the high-affinity ACh-bound state. This further suggests that G_i1_ is a superior PAM compared to LY211 because no further modulation was observed. However, the addition of LY211 did promote a further collapse in [^3^H]-NMS binding, suggesting an allosteric interaction between the G protein and the PAM for the antagonist-bound conformation. Given that LY211 and G_i1_ displayed similar cooperativity values for [^3^H]-NMS binding, it is not unreasonable to think that the NAM effect could be additive. To explore more accurately, we titrated a wider range of LY211 concentrations against a *K*_D_ concentration of [^3^H]-NMS with and without a saturating amount of G_i1_ and determined the affinity (p*K*_B_) and log α_[3H]-NMS_ of LY211 through the use of the ATCM (Fig. 3C and Table 1). Without G protein, the affinity and cooperativity values resembled those obtained in the full interaction between [^3^H]-NMS, ACh, and LY211, as expected. In the presence of G protein, a sixfold increase in the affinity of LY211 was observed and an increase in the ability of LY211 to allosterically displace [^3^H]-NMS (Fig. 3D and Table 1). Compared to the log α_[3H]-NMS_ of G protein only (fig. S6A and Table 1), this decrease when G protein and LY211 are combined indicates an additive NAM effect and additional destabilization of the inactive state.

**Fig. 4.**
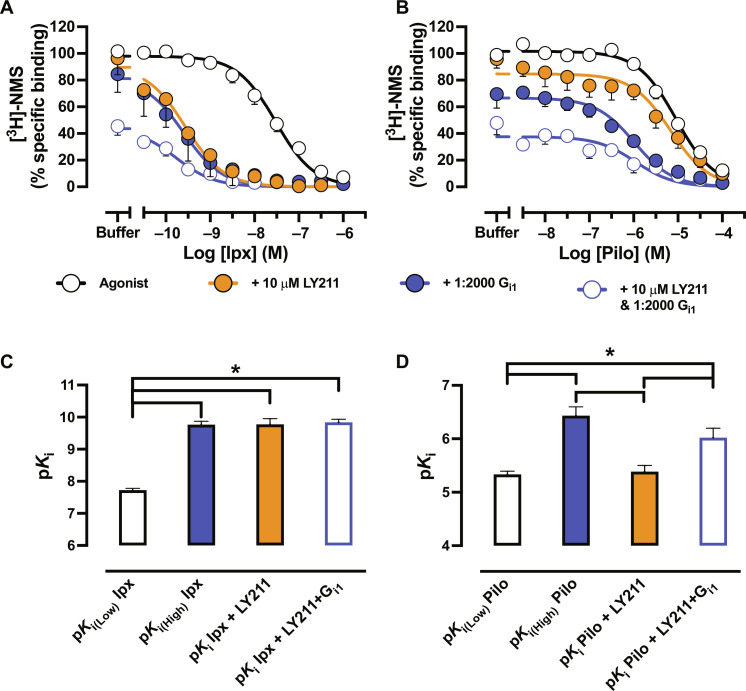
Modulating the low- and high-affinity state of super and partial agonists by G protein and PAM. Radioligand competition binding between (**A**) Ipx or (**B**) Pilo with [^3^H]-NMS at M_2_ mAChR nanodiscs in the presence of 10 μM LY2119620, a saturating stoichiometric 1:2000 ratio of receptor to G_i1_ proteins, or 10 μM LY2119620 plus a saturating stoichiometric 1:2000 ratio of receptor to G_i1_ proteins. Data were normalized to the buffer-only condition of Ipx or Pilo. A one-state model of competition binding was globally fit to the data. Low- and high-affinity values of (**C**) Ipx were obtained in (A), and (**D**) Pilo was obtained in (B). For all panels, data represent the means ± SEM of at least three individual experiments performed in duplicate. Group sizes and obtained parameters are listed in Table 1. *, significantly different, *P* < 0.05, one-way ANOVA, Tukey’s multiple comparisons test.

Because LY211 is a Ago-PAM that can promote the active state of the receptor on its own (Fig. 1B) (*24*, *42*), we investigated what influence LY211 had on the high-affinity state of Ipx or Pilo. Particularly in the case of Pilo where low cooperativity values were observed for both G protein and PAM, we investigated if the presence of both modulators would enhance Pilo’s ability to partially shift the AR to AR* receptor equilibrium. Because LY211 did not enhance the ACh-bound high-affinity state (Fig. 3B), we measured the affinity of Ipx or Pilo in the presence of G protein without [p*K*_i(High)_] and with [p*K*_i(High+PAM)_] 10 μM LY211 (blue open and blue closed circles, respectively, Fig. 4, A to D). In this way, we could also perform (i) a competition binding curve of Ipx or Pilo without G protein [p*K*_i(Low)_; black open circles, Fig. 4, A to D] and (ii) Ipx and Pilo without G protein but with 10 μM LY211 [p*K*_i(Low+PAM)_; orange circles, Fig. 4, A to D] in parallel. By performing the experiment in this manner, the influence that PAMs and G proteins have on agonist and [^3^H]-NMS affinity is more appropriately determined and compared. The presence of 10 μM LY211 significantly increased the affinity of Ipx (112-fold), similar to the influence of G protein on Ipx affinity (Fig. 4, A and C, and Table 1). Adding both G protein and LY211 produced no significant increases in Ipx affinity beyond what either the G protein alone or PAM alone produced. In the case of Pilo, a nonsignificant increase in affinity was observed in the presence of 10 μM LY211. Similar to ACh and Ipx, the p*K*_i(High)_ of Pilo was not altered in the presence of PAM (Fig. 4, B and D, and Table 1). Thermodynamically, this indicates that the inherent efficacy of the orthosteric agonist in combination with the G protein, as a superior PAM, determines the formation of the high-affinity state and that small-molecule PAMs cannot increase orthosteric ligand affinity once this high-affinity state is formed.

### Kinetic stabilization of the high-affinity state by PAMs

**Fig. 5.**
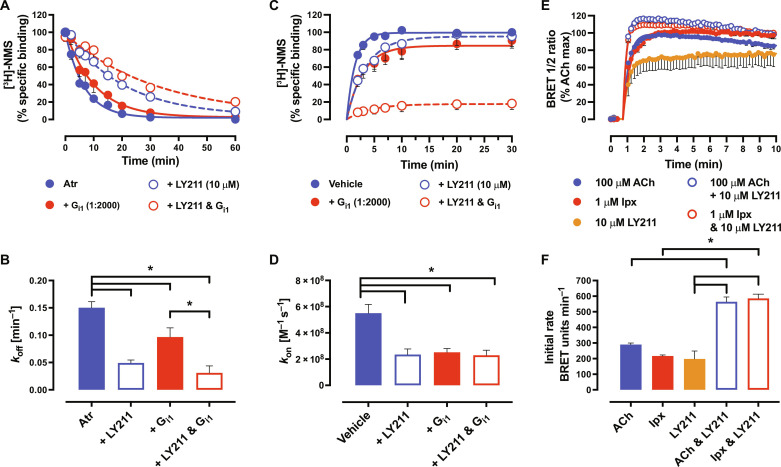
PAMs kinetically stabilize the high-affinity state of the ternary complex. (**A**) [^3^H]-NMS dissociation at M_2_ mAChR nanodiscs in the presence of Atr, Atr + 10 μM LY2119620, Atr + a saturating amount of G_i1_, and Atr + 10 μM LY2119620 + a saturating amount of G_i1_. Data were normalized to time point zero. A one-phase exponential decay dissociation model was globally fit to the data. (**B**) Dissociation rates (*k*_off_, min^−1^) of data presented in (A). (**C**) [^3^H]-NMS association at M_2_ mAChR nanodiscs in the presence of Atr, Atr + 10 μM LY2119620, Atr + a saturating amount of G_i1_, and Atr + 10 μM LY2119620 + a saturating amount of G_i1_. Data were normalized to time point zero. A one-phase exponential decay dissociation model was globally fit to the data and constrained to *k*_off_ values obtained in (A). (**D**) Association rates (*k*_on_, M^−1^ s^−1^) of data presented in (C). (**E**) Kinetic G_i1_ protein activation at M_2_ mAChR FlpIn CHO cells transiently transfected with TRUPATH sensors in response to saturating concentrations of orthosteric and allosteric ligands. Data were baseline corrected to a buffer-only condition and normalized to ACh only, and a “baseline then rise to steady state with drift” model was fit to the data. (**F**) Initial rate values, *v_o_*, of data presented in (E). For (A) to (F), data represent the means ± SEM of at least three individual experiments performed in duplicate. Group sizes and obtained parameters are listed in Table 1. *, significantly different, *P* < 0.05, one-way ANOVA, Tukey’s multiple comparisons test.

To further investigate the effect of PAMs on the high-affinity state, we turned to dissociation kinetic experiments to measure any kinetic influence the PAMs had on the high-affinity state. We preincubated the receptor with a radioligand and added a saturating concentration of the orthosteric ligand atropine (Atr) to prevent rebinding of the radioligand, together with LY211 and/or G protein at different time points. Formation of the active state (through the addition of PAM or G protein) is expected to trap [^3^H]-NMS (*17*, *43*), leading to a decrease in the dissociation rate of the radioligand. The extent of ternary complex stabilization by PAM or G protein will therefore be reflected in changes in [^3^H]-NMS dissociation rates. Without G protein and in line with previous results (*42*), LY211 impeded the dissociation of the orthosteric radioligand (Fig. 5, A and B, and Table 1). Measuring dissociation in the presence of a saturating amount of G protein also led to a decrease in the dissociation rate of [^3^H]-NMS, consistent with a previous study that measured the impact of G_s_ on antagonist dissociation at the β_2_AR (*17*). Notably, combining both PAM and G protein produced a further decrease in the dissociation rate of the orthosteric radioligand (Fig. 5, A and B, and Table 1), suggestive of a synergistic closing of the orthosteric pocket where the PAM and G protein combine to facilitate the transition of receptors to the fully closed, active state. Given that equilibrium affinity is a composite of kinetic association and dissociation rates and no changes in p*K*_i(High)_ at the M_2_ mAChR were seen with the addition of PAM, one would expect the association rate of an orthosteric ligand at the ternary complex to decrease in the presence of PAM. We preincubated the receptor with PAM and/or G protein and then added a radioligand at different times. Encouragingly, the G protein or PAM alone caused a decrease in the observed association rate of [^3^H]-NMS. The combination of both led to a large collapse and slowing down of the binding of [^3^H]-NMS such that, over the timescales used, a large proportion of receptors were precluded from radioligand binding (as evidenced by a decrease in *B*_max_) (Fig. 5, C and D, and Table 1). Together, these findings suggest a synergistic kinetic stabilization of the active ternary complex by PAM and G protein.

### Impact of ternary complex stabilization on the rate of initial signaling

To determine if the kinetic impact of the PAM on the ternary complex influences G protein activation, we turned to measuring the kinetics of G protein activation using the BRET TRUPATH assay in M_2_ mAChR FlpIn CHO cells. Using a recently described GPCR kinetic signaling model (*44*, *45*), we derived an initial rate (*v_o_*) of signaling for saturating concentrations of ACh, Ipx, LY211 alone, and ACh or Ipx in combination with LY211. The term *v_o_* is commonly used in enzyme kinetics and measures the rate of product formation before substrate degradation or establishment of equilibrium (the slope of the straight line of an activation curve). For a GPCR in a TRUPATH assay, this represents the efficiency of the agonist(s)-occupied receptor at promoting G protein activation in the absence of any regulation or desensitization mechanisms and can therefore be considered a direct reflection of the extent of active-state stabilization by the ligand(s).

ACh and Ipx displayed similar G protein activation kinetics (Fig. 5, E and F, and Table 1). Conversely, LY211 displayed slower G protein activation kinetics. However, when combining ACh and Ipx with LY211, a more rapid G protein activation was observed. A similar trend was observed in steady-state values [which represent the final effect level stimulated by the ligand(s)] as ACh and Ipx displayed similar values that were both enhanced by LY211 (Fig. 5, E and F, and Table 1). Together, this indicates that the increased stabilization of the ternary complex in the presence of an orthosteric agonist and PAM leads to more efficient and rapid G protein activation.

## DISCUSSION

GPCRs are inherently allosteric proteins owing to the distal communication that is required between the binding of an extracellular ligand and the activation of an intracellular transducer partner. Previous studies have shown that the allosteric coupling between G protein and orthosteric agonists leads to the formation of the ternary complex and high-affinity state that is structurally and pharmacologically characterized by the closing of the orthosteric pocket and increased orthosteric agonist affinity, respectively (*17*, *24*). PAMs bind to a secondary ligand binding site, distinct from the endogenous ligand site, through which an allosteric transition enhances orthosteric agonist affinity. How a PAM influences the ternary complex and high-affinity state and the impact this has on GPCR signaling have remained largely unknown.

Through radioligand binding experiments with purified M_2_ mAChR nanodiscs and purified heterotrimer G protein, we show that, once the high-affinity state is formed, the PAM LY211 does not promote a further increase in agonist affinity. This indicates that, irrespective of orthosteric agonist efficacy, the agonist and G protein alone form the high-affinity state. The observation that a G protein is a superior PAM of agonist affinity compared to an exogenous PAM (LY211) may reflect the requirement of an allosteric link between the orthosteric binding site and G protein for GPCR activation and signaling. Our radioligand kinetic experiments show that the PAM LY211 stabilizes the lifetime of the ternary complex. Nanodiscs provide a suitable platform for the interrogation of the ternary complex due to the ability to control the presence of nucleotides and the concentration of each component. However, in a physiological setting, the ternary complex is transient due to the presence of freely available nucleotide. Measuring the kinetics of G protein activation in a recombinant cell environment and deriving a *v_o_* parameter that represents the efficiency of the agonist-bound receptor (i.e., active-state receptor), signaling parameters that capture agonist activity at the transient ternary complex were determined. These results show that PAMs promote the ability of orthosteric agonists to activate the G protein and, together with the observations from the nanodisc radioligand dissociation kinetics, indicate that this arises from kinetic stabilization of the ternary complex that leads to more efficient G protein binding and activation (Fig. 5, E and F).

The discovery and development of allosteric modulators require the appropriate analysis and pharmacological quantification of parameters that capture the influence of allosteric modulators on orthosteric ligands and GPCR signaling. Through thermodynamic models, parameters reflective of equilibrium can be captured, including the ATCM that quantifies affinity (p*K*_B_) and binding cooperativity (log α) (*7*, *41*) and the operational model of allosterism that quantifies functional cooperativity (log αβ) and allosteric agonism (log τ) (*32*). A kinetic ATCM has been developed to account for nonequilibrium artifacts in binding experiments due to the ability of allosteric modulators to slow the ligand-receptor interaction (*46*, *47*). There is a growing appreciation for the kinetic aspects of ligand binding and GPCR signaling (*48*–*51*) with numerous studies linking ligand kinetics (*52*, *53*) of orthosteric agonists to increased signaling (*54*, *55*), biased agonism (*56*, *57*), and the dynamics of conformational changes (*58*). However, the kinetic influence of allosteric modulators on GPCR signaling has remained relatively unexplored. Here, we have begun to explore this concept by using a recently developed GPCR signaling kinetics model to show that PAMs enhance the initial rate of signaling at an orthosteric agonist–occupied receptor. Ultimately, this knowledge can be used to explore further the influence of allosteric modulators on GPCR signaling kinetics.

In the case of the adenosine A_1_ receptor (A_1_AR) reconstituted into nanodiscs, the PAM MIPS521 also slowed the dissociation rate of radioligand in the presence of G protein representative of ternary complex stabilization (*43*). Comparatively, the allosteric binding site of LY211 sits directly above and caps the orthosteric binding site, while the binding site of MIPS521 at the A_1_AR is distal to the orthosteric binding site, sitting outside of the receptor at the lipid interface (fig. S7). These findings suggest a similar mechanism for stabilizing the high-affinity state across class A GPCRs irrespective of the position of the allosteric binding site. Differences exist between MIPS521 and LY211 in their ability to impede radioligand dissociation. For example, MIPS521 only impedes radioligand dissociation at A_1_AR nanodiscs in the presence of G protein, whereas LY211 can retard radioligand dissociation in both the presence and absence of G proteins. This can be rationalized by the differences in their binding site loci as the LY211 allosteric binding site is located directly above the orthosteric binding site, which sterically blocks orthosteric ligand dissociation. MIPS521, on the other hand, impedes dissociation solely by stabilizing the high-affinity state as its allosteric binding site is distal to the orthosteric binding site.

Although nanodiscs provide a reductionist environment where the high-affinity state can be studied by ensuring the correct stoichiometry of receptor to G protein, they are an artificial system where the complex interactions present in the cellular milieu are lost. Agonist-receptor interactions that are influenced by, for example, homo-/hetero-oligomers, posttranslational modifications, membrane composition, endogenous allosteric modulators, and different transducers or regulatory proteins are thus not accounted for within a nanodisc system. Understanding the impact of these processes and others on agonist high-affinity binding will provide a more complete snapshot of the ternary complex and signal transduction. The use of a dominant negative G protein with reduced nucleotide affinity (*59*) is an alternative strategy to explore high-affinity binding in a cellular environment, as recently done at the metabotropic glutamate 2 (mGlu2) where it was shown to enhance agonist potency (*60*).

In our study, the ability of allosteric modulators to influence orthosteric ligand binding was explored experimentally through radioligand binding with an antagonist radioligand and conceptually through an ATCM that both bring inherent limitations. Experimentally, the antagonist radioligand prevents the formation of active state that the agonist, PAM, and G protein is promoting. However, studies at the β_2_AR have shown that G protein–mediated stabilization of the active state can trap antagonist radioligands (*17*, *61*) and our own kinetic association data suggest that G protein traps [^3^H]-NMS (Fig. 5C). Future studies with the use of an agonist radioligand can build on our findings where all components within the assay facilitate active-state stabilization. Conceptually, within the ATCM framework, there is only one inactive-state (low-affinity binding) and one active-state (high-affinity binding) conformation. Although most known allosteric modulators at the mAChRs operate through a two-state model of allosterism (*22*), questions remain over different forms of allosteric modulation. One such example is biased modulation, where allosteric modulators change the relative signaling capacity of an orthosteric agonist in one pathway over others (*62*). Numerous examples of biased modulation exist, and it is thought to occur through the presence of multiple agonist-bound active states, each linked to a different functional output, where the allosteric modulator changes the relative abundance of these active states (*63*). At the M_2_ mAChR, analyzing conformational dynamics through the use of ^13^CH_3_-ε-methionine NMR, it was shown that the M_2_ mAChR is highly dynamic and exhibits a wide range of receptor conformations (*64*) that are altered in the presence of LY211 (*65*). Similarly, at class C GPCRs, the mGlu2 receptor exists in a number of conformational states when analyzed through single-molecule Förster resonance energy transfer (smFRET) using fluorophores linked to SNAP tags in a detergent micelle environment (*66*). The presence of an agonist increased the fraction of receptors in the active state, and consistent with the results presented here, this approach found that the addition of a PAM led to increased stabilization of the active state. This study also showed that the combined effect of PAM and G protein was not additive (*66*). A follow-up study focusing on the Venus flytrap (VFT) domain indicated that PAMs stabilize the closed, active state of the VFT that is induced by agonists (*67*), while a similar smFRET approach showed that the dynamics of the 7 transmembrane and cysteine-rich domain were stabilized when the mGlu2 is co-bound to both an agonist and PAM (*68*). In contrast to our Ago-PAM LY211, these studies at the mGlu2 were performed with a pure PAM that does not display agonism in its own right and only displays active-state stabilization in the presence of orthosteric agonists. Our results thus extend this mechanism of action for a Ago-PAM and further verify these findings at class A GPCRs using radioligand binding experiments with receptors purified into a more native nanodisc environment. Furthermore, we extend this knowledge by linking the active-state stabilization of PAMs to increases in the signaling efficiency of the ternary complex in a recombinant cell environment. By tying together the thermodynamic and kinetic impacts of PAM and G protein on stabilizing the active receptor state through a novel combination of biochemical and analytical approaches, our results provide further evidence of the molecular mechanisms that govern positive allosteric modulation at GPCRs.

## MATERIALS AND METHODS

### Materials

Dulbecco’s modified Eagle’s medium (DMEM) and CHO FlpIn cells were purchased from Invitrogen. Fetal bovine serum (FBS) was purchased from Thermotrace (Melbourne, Australia). Hygromycin B was purchased from Roche Applied Science. [^3^H]-NMS (specific activity, 70 Ci/mmol) and MicroScint-O were purchased from PerkinElmer Life and Analytical Sciences (Waltham, MA, United States). The AlphaScreen-based Sure-Fire cellular ERK1/2 assay kit was purchased from TGR BioSciences (Adelaide, Australia). Prolume Purple was purchased from NanoLight Technology (Pinetop, AZ, United States). All other chemicals were purchased from Sigma-Aldrich Chemical Company (St. Louis, MO, United States).

### Mammalian tissue culture

FlpIn CHO cells stably expressing mAChR constructs were cultured at 37°C in 5% CO_2_ using DMEM supplemented with 5% (v/v) FBS. Upon reaching confluence, media were removed, and cells were washed with phosphate-buffered saline (PBS) and harvested from tissue culture flasks using Versene. Cells were then centrifuged at 350*g* for 3 min, followed by resuspension in DMEM + 5% FBS. Cells were then either plated for an assay or reseeded into a tissue culture flask.

### ERK1/2 phosphorylation assay

The AlphaScreen-based SureFire kit was used to measure phosphorylated ERK1/2 (pERK1/2). M_2ΔICL3_ mAChR FlpIn CHO cells were plated into 96-well plates at a density of 25,000 cells per well and incubated overnight at 37°C. The following day, the growth medium was replaced with serum-free DMEM for a minimum of 6 hours at 37°C. The cells were stimulated with a range of concentrations of drugs for an incubation period that matched the peak response time of the drug. Peak response time was determined by measuring pERK1/2 response at a range of time points over a 30-min period. 10% FBS (v/v) was used as a positive control. Media were flicked off, and cells were lysed with SureFire lysis buffer (80 μl per well) and stored at −20°C overnight. Plates were thawed at RT, and 10 μl of the cell lysates was transferred to a 384-well OptiPlate. Under reduced lighting conditions, 8.5 μl of detection buffer (buffer consisting of reaction buffer/activation buffer/acceptor beads/donor beads in a 60:10:0.3:0.3 ratio) was added, and plates were incubated for 1 hour at 37°C. The fluorescence signal was measured using an EnVision multilabel plate reader (PerkinElmer) with AlphaScreen settings. Data were expressed as a percentage of the pERK1/2 mediated by 10% FBS.

### G protein activation assay

Upon 60 to 80% confluence, WT hM_2_ mAChR expressing FlpIn CHO cells were transfected transiently using polyethylenimine (Sigma-Aldrich) with 10 ng per well of each the G protein TRUPATH biosensors pcDNA5/FRT/TO-Gα_i1_-RLuc8/pcDNA3.1-β_3_/pcDNA3.1-Gγ_9_-GFP2 giving a ratio of 1:1:1 ratio with 30 ng in total. Cells were plated at 30,000 cells per well into 96-well Greiner CELLSTAR white-walled plates (Sigma-Aldrich). Forty-eight hours later, cells were washed with 200 μl of PBS and replaced with 1x Hanks’ balanced salt solution supplemented with 10 mM Hepes. Cells were incubated for 30 min at 25°C before addition of 10 μl of 1.3 μM Prolume Purple coelenterazine (NanoLight Technology, Pinetop, AZ). Cells were further incubated for 10 min at 25°C before BRET measurements were performed on a PHERAstar FSX plate reader (BMG Labtech) using 410/80-nm and 515/30-nm filters. Four baseline measurements for each well were taken before the addition of drugs or vehicle to give a final assay volume of 100 μl, and ligand-induced changes in BRET measurements were then taken for an additional 10 min. The BRET signal was calculated as the ratio of 515/30-nm emission over 410/80-nm emission. The ratio was vehicle corrected using the initial four baseline measurements and then baseline corrected again using the vehicle-treated wells. Concentration-response curves were constructed from the area under the curve (AUC) of the double baseline-corrected kinetic traces and normalized to the highest AUC value in each dataset. For kinetic TRUPATH experiments, the ligands were added manually after four baseline reads, and each experiment was performed with a maximum of six wells to avoid a time lag between reads: two buffer-only wells, two ACh *E*_max_ wells, and two treatment wells. The double baseline-corrected BRET ratios were normalized to the greatest ACh-induced BRET decrease in each dataset.

### Membrane preparation

At confluence, WT hM_2_ mAChR FlpIn CHO cells were harvested and pelleted. The pellet was resuspended in Hepes homogenization buffer (50 mM Hepes, 2.5 mM MgCl_2_, and 2 mM EGTA) and homogenized for three 10-s intervals with a 30-s cooling interval on ice. The homogenate was centrifuged for 10 min at 600*g*, and the supernatant was stored on ice. The remaining pellet was resuspended in Hepes homogenization buffer, and the homogenization process was repeated until no further reduction in pellet size occurred following centrifugation. The supernatant was placed into centrifugation tubes and centrifuged (30,000*g*, 30 min, 4°C), and the pellet was resuspended into binding buffer (20 mM Hepes, 100 mM NaCl, and 10 mM MgCl_2_). Protein concentration was determined using the Pierce BCA protein assay kit (Thermo Fisher Scientific).

### ApoA1 cMSP1D1 expression and purification

*Escherichia coli* BL21 (DE3) cells were transformed with split intein cMSP1D1 constructs inserted into a pET28a vector (Novagen) in LB media + kanamycin. Induction of protein expression was accomplished through 500 mM isopropyl-β-d-1-thiogalactopyranoside at an OD600 (optical density at 600 nm) of 0.6, and cells were shaken for 16 to 20 hours at 25°C. Cells were harvested by centrifugation (7000*g*, 20 min, 4°C), the cell pellet was resuspended in lysis buffer [50 mM Tris (pH 8.0), 250 mM NaCl, and 0.5% Triton X-100], 0.5 mM EDTA, and 1 mM phenylmethylsulfonyl fluoride (PMSF), and the cells were lysed by incubation with lysozyme (50 μg/ml) for 30 min and further sonication. The lysate was dounced and put through Avestin followed by a 30-min incubation at 4°C with 2 μl benzonase + 10 μM benzamidine and 5 mM MgCl_2_. Cell debris were removed by centrifugation (30,000 *g*, 30 min, 4°C). A heat shock at 70°C was conducted with the soluble fraction for 40 min. Aggregates were removed by centrifugation (30,000*g*, 30 min, 4°C). The supernatant was loaded onto a gravity-flow Ni-NTA resin column (GE HealthCare). Flow-through and 1 column volume (CV) of wash buffer [20 mM Tris (pH 8.0), 320 mM NaCl, 10 mM imidazole, and 10 mM 2-mercaptoethanol (BME)] were collected and dialyzed to 20 mM Tris (pH 8.0), 0.5 mM EDTA, and 10 mM BME. The urea concentration was set to 6 M, and the sample was applied to a 5-ml HiTrap QFF anion-exchange column (GE HealthCare) and was eluted using a 30-CV-long gradient from low salt buffer [20 mM Tris (pH 8.0), 0.5 mM EDTA, 6 M urea, and 10 mM BME] to high salt buffer [20 mM Tris (pH 8.0), 300 mM NaCl, 0.5 mM EDTA, 6 M urea, and 10 mM BME]. The pure protein was pooled, dialyzed to 20 mM Tris (pH 8.0), 200 mM NaCl, 0.5 mM EDTA, and 10 mM BME, concentrated using a 10-kDa molecular mass cutoff centrifugal filter unit (Millipore, Burlington, MA, United States), flash frozen using liquid nitrogen, and stored at −80°C.

### M_2_ mAChR expression and purification

The human M_2_ muscarinic receptor gene (http://cdna.org) was modified to give a receptor containing an N-terminal anti-Flag epitope tag and a C-terminal 8× histidine tag. To increase stability and expression, residues 226 to 389 of intracellular loop 3 (ICL3) were removed. The resulting Flag-M_2ΔICL3_-His construct was cloned into a pFastbac baculovirus transfer vector, and the Bac-to-Bac Baculovirus Expression System (Invitrogen) was used to generate a baculovirus. The M_2_ mAChR protein was expressed using baculovirus-infected Sf9 cells. Cells were grown in ESF 921 serum-free media (Expression Systems) and infected at a density of 4.0 × 10^6^ cells/ml, treated with 10 μM Atr, and shaken at 27°C for 48 to 60 hours. Cells were harvested by centrifugation (10,000*g*, 20 min, 4°C), and the cell pellet was resuspended in lysis buffer [10 mM Tris (pH 7.5), 1 mM EDTA, 1 mM MgCl_2_, protease inhibitors (500 μM PMSF, 1 mM leupeptin-trypsin inhibitor, and 1 mM benzamidine), iodoacetamide (1 mg/ml), benzonase, and 1 μM Atr] and stirred at 25°C until homogeneous. The cell lysate was centrifuged (15,000 rpm, 15 min, 4°C). The receptor was solubilized in solubilization buffer [30 mM Hepes (pH 7.5), 1% *n*-dodecyl β-d-maltoside (DDM), 0.2% cholate, 0.03% cholesterol hemisuccinate (CHS), 750 mM NaCl, 30% glycerol, protease inhibitors (500 μM PMSF, 1 mM LT, and 1 mM benzamidine), iodoacetamide (1 mg/ml), benzonase, and 1 μM Atr]. The soluble fraction was separated through centrifugation (15,000 rpm, 15 min, 4°C), and the supernatant was incubated with the Ni-NTA resin for 2 hours at 4°C. The Ni-NTA resin was washed with wash buffer [30 mM Hepes (pH 7.5), 0.1% DDM, 0.02% sodium cholate, 0.003% CHS, 750 mM NaCl, 30% glycerol, 5 mM imidazole, and 1 μM Atr), and the protein was eluted with wash buffer supplemented with 250 mM imidazole. The sample was loaded onto an M1 anti-Flag affinity resin, and the detergent was exchanged from DDM solubilization buffer to lauryl maltose neopentyl glycol (LMNG) buffer [30 mM Hepes (pH 7.5), 0.01% LMNG, 0.001% CHS, 100 mM NaCl, and 1 μM NaCl]. Protein was eluted off an M1 anti-Flag affinity resin with LMNG buffer supplemented with 10 mM EDTA and Flag peptide (0.2 mg/ml). Elution was concentrated and run through SEC using a Superdex200 Increase 10/300 column (GE HealthCare) with LMNG buffer. The sample was collected, concentrated, and flash frozen using liquid nitrogen, and stored at −80°C.

### G protein expression and purification

WT G protein α subunits were cloned into a pVL1392 baculovirus transfer vector. G protein β and γ subunits were cloned into a pVL1392 baculovirus transfer vector with the β subunit modified to contain a C-terminal 8× histidine tag. Baculovirus was generated using the pVL1392 constructs and BestBac Linearized DNA (Expression Systems). G protein subunits were expressed using baculovirus-infected *Trichoplusia ni* (Hi5) insect cells. Cells were grown in ESF 921 serum-free media (Expression Systems) and infected at a density of 4.0 × 10^6^ cells/ml with a 1:1 ratio of Gα to Gβγ viruses and shaken at 27°C for 48 to 60 hours. Cells were harvested by centrifugation (10,000*g*, 20 min, 4°C), and the cell pellet was lysed in lysis buffer [10 mM Tris (pH 7.4), 5 mM MgCl_2_, 5 mM tris(2-carboxyethyl)phosphine (TCEP), 10 μM guanosine 5′-diphosphate (GDP), protease inhibitors (500 μM PMSF, 1 mM leupeptin, and 1 mM benzamidine), and benzonase]. The cell lysate was centrifuged (18,000 rpm, 15 min, 4°C). The cell pellet was solubilized in 20 mM Hepes (pH 7.5), 100 mM NaCl, 1.0% sodium cholate, 0.05% DDM, 5 mM MgCl_2_, 1 mM TCEP, 10 μM GDP, protease inhibitors (500 μM PMSF, 1 mM LT, and 1 mM benzamidine), and 20 mM imidazole and stirred for 60 min at 4°C followed by centrifugation (18,000 rpm, 15 min, 4°C). The filtered supernatant was incubated with the Ni-NTA resin for 90 min at 4°C. The resin was loaded onto a glass column and washed with wash buffer [20 mM Hepes (pH 7.5), 100 mM NaCl, 0.05% DDM, 1 mM MgCl_2_, 1 mM TCEP, 10 μM GDP, protease inhibitors (500 μM PMSF, 1 mM LT, 1 mM benzamidine), and 20 mM imidazole], until no more protein was coming off as determined by Bradford. The sample was eluted with wash buffer + 250 mM imidazole and was dialyzed overnight at 4°C to remove imidazole and to lower NaCl to 50 mM. The following morning, the sample was loaded onto a 5-ml HiTrap QFF anion-exchange column (GE HealthCare) and then washed with 15 CV of buffer A [20 mM Hepes (pH 7.4), 25 mM NaCl, 0.1% DDM, 1 mM MgCl_2_, 100 μM TCEP, and 10 μM GDP]. A gradient of 0 to 30% over 20 CV was then run with buffer A and buffer B [20 mM Hepes (pH 7.4), 1 M NaCl, 0.1% DDM, 1 mM MgCl_2_, 100 μM TCEP, and 10 μM GDP]. Samples were collected and diluted with 20 mM Hepes (pH 7.4), 30 mM NaCl, 0.1% DDM, 1 mM MgCl2, 100 μM TCEP, and 10 μM GDP to dilute NaCl to a final concentration of 125 mM. The sample was concentrated, glycerol added to 20%, flash frozen using liquid nitrogen, and stored at −80°C.

### Incorporating M_2_ mAChR into nanodiscs

1-Palmitoyl-2-oleoyl-*sn*-glycero-3-phosphocholine (POPC; Avanti Polar Lipids) and 1-palmitoyl-2-oleoyl-*sn*-glycero-3-phospho-(1′-*rac*-glycerol) (POPG; Avanti Polar Lipids) were mixed using chloroform to a final concentration of 10 mM POPC and 6.67 mM POPG (3:2 POPC:POPG ratio). The lipid mixture was dried using nitrogen to evaporate the chloroform; residual moisture and any remaining chloroform were then removed using an overnight incubation step in a desiccator. The following morning, the lipid mixture was resuspended in HNE buffer [20 mM Hepes (pH 8.0), 100 mM NaCl, 1 mM EDTA, and 50 mM sodium cholate] and sonicated on ice until the lipid solution became clear. The lipid mixture was flash frozen using liquid nitrogen and stored under nitrogen at −80°C. To determine the optimum ratio of lipid to ApoA1, different ratios of ApoA1 to lipid (1:40, 1:50, 1:60, 1:70, and 1:80) were tested with 100 μM ApoA1 to determine the optimum ratio that created a homogeneous, monodisperse sample as determined by SEC using a Superdex200 Increase 10/300 column (GE HealthCare). In brief, the appropriate amount of lipid mixture, HNE buffer [20 mM Hepes (pH 8.0), 100 mM NaCl, and 1 mM EDTA], and 100 μM ApoA1 in a 100-μl volume was mixed together. Following incubation on ice for 1 hour, the mixture was incubated with ~50 mg of Bio-Beads (Bio-Rad) to remove all detergents and initiate the spontaneous formation of nanodiscs. The sample was mixed overnight at 4°C, and the following morning, the nanodiscs were purified using SEC on a Superdex200 Increase 10/300 column (GE HealthCare) with HNE buffer. To incorporate the purified receptor, the appropriate lipid ratio was chosen, a 1:60 ratio of cMSP1D1 to POPC:POPG, and nanodisc reconstitution was performed as described above with the inclusion of a 5 μM purified receptor. Samples were pooled, flash frozen using liquid nitrogen, and stored at −80°C. For nanodiscs that were reconstituted with M_2_ mAChR purified with Atr, the sample was dialyzed for 24 hours at 4°C with PBS prior to flash freezing to remove residual Atr.

### Fab complexing

The Pierce Fab Preparation Kit (Thermo Fisher Scientific) generated Fab fragments of anti-Flag immunoglobulin G antibody. Purified Fab fragments were added to M_2_ mAChR cMSP1D1 at a ratio of 10:1, where the concentration of the receptor in the nanodisc was calculated through saturation radioligand binding with [^3^H]-NMS (described in general methods). The sample was incubated with 5 mM MgCl_2_ and incubated at RT for 4 hours. Following incubation, the sample was loaded over 0.5 ml of Ni resin, and flow-through was collected and reloaded for a total of five times. The resin was washed until no more protein was coming off the column, as determined by Bradford, and the sample was eluted with PBS + 250 mM imidazole. Samples were concentrated and analyzed through negative staining.

### Radioligand binding

The affinity of [^3^H]-NMS for M_2_ mAChR in nanodiscs, as well as the concentration of receptor in nanodiscs, was determined through saturation binding with [^3^H]-NMS. M_2_ nanodiscs were incubated with a range of concentrations of [^3^H] NMS in a final volume of 200 μl of binding buffer [20 mM Hepes (pH 7.5), 100 mM NaCl, 10 mM MgCl_2_, and 0.5% bovine serum albumin] for 6 hours at RT. To determine the low and high affinity of orthosteric agonists at M_2_ mAChR nanodiscs, nanodiscs were incubated in a final volume of 200 μl of binding buffer containing a range of concentrations of the cold ligand in the presence of a *K*_D_ concentration of [^3^H]-NMS as determined through saturation binding in the presence of a saturating/increasing stoichiometric amount of G protein for 6 hours at RT. The concentrated G protein was added such that DDM and GDP were diluted at least 1600-fold. For interaction experiments with LY211, competition binding between a *K*_D_ concentration of [^3^H]-NMS and a range of concentrations of an orthosteric drug was performed in the presence of varying concentrations of LY211 with or without a saturating amount of purified G protein. For dissociation experiments, M_2_ mAChR nanodiscs were incubated with a *K*_D_ concentration of [^3^H]-NMS for 1 hour at RT in a total volume of 200 μl of binding buffer. Dissociation of the radioligand was initiated by addition of 10 μM Atr alone or in the presence of LY211 and G protein at various time points. For association experiments, M_2_ mAChR nanodiscs were incubated with LY211 and G protein for 4 hours at RT before the association was initiated through the addition of a *K*_D_ concentration of [3H]-NMS at various time points. Membrane equilibrium binding experiments were performed with 15 μg of membrane expressing the WT hM_2_ mAChR in a 300-μl reaction volume of binding buffer with or without 100 μM Gpp[NH]pp in deep 96-well plates for 6 hours at RT. Membranes were incubated with a range of concentrations of ACh in the presence of a *K*_D_ concentration of [^3^H]-NMS as determined through saturation binding. For all radioligand binding experiments, nonspecific binding was defined by 10 μM Atr and the assay was terminated through rapid filtration through Whatman GF/B filter plates using a 96-well harvester (PerkinElmer). Filter plates were dried overnight, and radioactivity was determined by adding 40 μl of MicroScint-O scintillation fluid and counting in a MicroBeta^2^ Plate Counter (PerkinElmer Life Sciences).

### Data analysis

All data were analyzed using GraphPad Prism 9 (GraphPad Software, San Diego, CA). Concentration-response curves were fit with a three-parameter logistic equation to quantify potency (pEC_50_) and an operational model of agonism to derive an efficacy parameter (log τ) (*69*). The interaction between ACh and LY211 in the G protein activation assay was fit to an operational model of allosterism to derive functional modulation (log αβ) and affinity (p*K*_B_) parameters (*32*). For competition binding experiments between [^3^H]-NMS and a range of concentrations of unlabeled orthosteric agonist at M_2_ mAChR nanodiscs in the presence of G protein and for competition binding at M_2_ mAChR membranes, data were fit to a two-site competition binding model or a one-site competition binding model. For the interaction of ACh with a range of LY211 concentrations with and without a saturating amount of G protein, data were fit to an ATCM to derive p*K*_B_ and α binding cooperativity parameters (*41*). For one-site and two-site competition models and ATCM, the affinity of NMS was constrained to the NMS value reflective of and corresponding to if M_2_ mAChR was bound to LY211, G_i1_, or LY211 + G_i1_. These [^3^H]-NMS affinity values for LY211, G_i1_, or LY211 + G_i1_ bound M_2_ mAChR were determined by adding the log α_[3H]-NMS_ cooperativity values of LY211, G_i1_, or LY211 + G_i1_ on [^3^H]-NMS binding reported in Table 1 from the affinity value of [^3^H]-NMS for unbound M_2_ mAChR. Monoexponential decay and one-phase association equations were fit to the radioligand dissociation and association data at M_2_ mAChR nanodiscs. Kinetic TRUPATH data were analyzed using a “baseline then rise to steady state with drift” equation (*44*). All affinity, potency, cooperativity, and efficacy parameters were estimated as logarithms, and statistical analyses between different treatment conditions were determined by one-way analysis of variance (ANOVA) using Tukey’s multiple comparisons with a value of *P* < 0.05 considered as significant.
