# Supplementary material for: Positive allosteric modulation of a GPCR ternary complex
Source: Sci Adv. 2024 Sep 11;10(37):eadp7040. doi: 10.1126/sciadv.adp7040 (PMC11389776; doi:10.1126/sciadv.adp7040)
Supplement: Supplementary file 1 — Figs. S1 to S7 [file sciadv.adp7040_sm.pdf]

Science Advances
AAAS

Supplementary Materials for

Positive allosteric modulation of a GPCR ternary complex

Wessel A. C. Burger *et al.*

Corresponding author: Celine Valant, celine.valant@monash.edu;
Arthur Christopoulos, arthur.christopoulos@monash.edu; David M. Thal, david.thal@monash.edu

Sci. Adv. **10**, eadp7040 (2024)
DOI: 10.1126/sciadv.adp7040

This PDF file includes:

Figs. S1 to S7

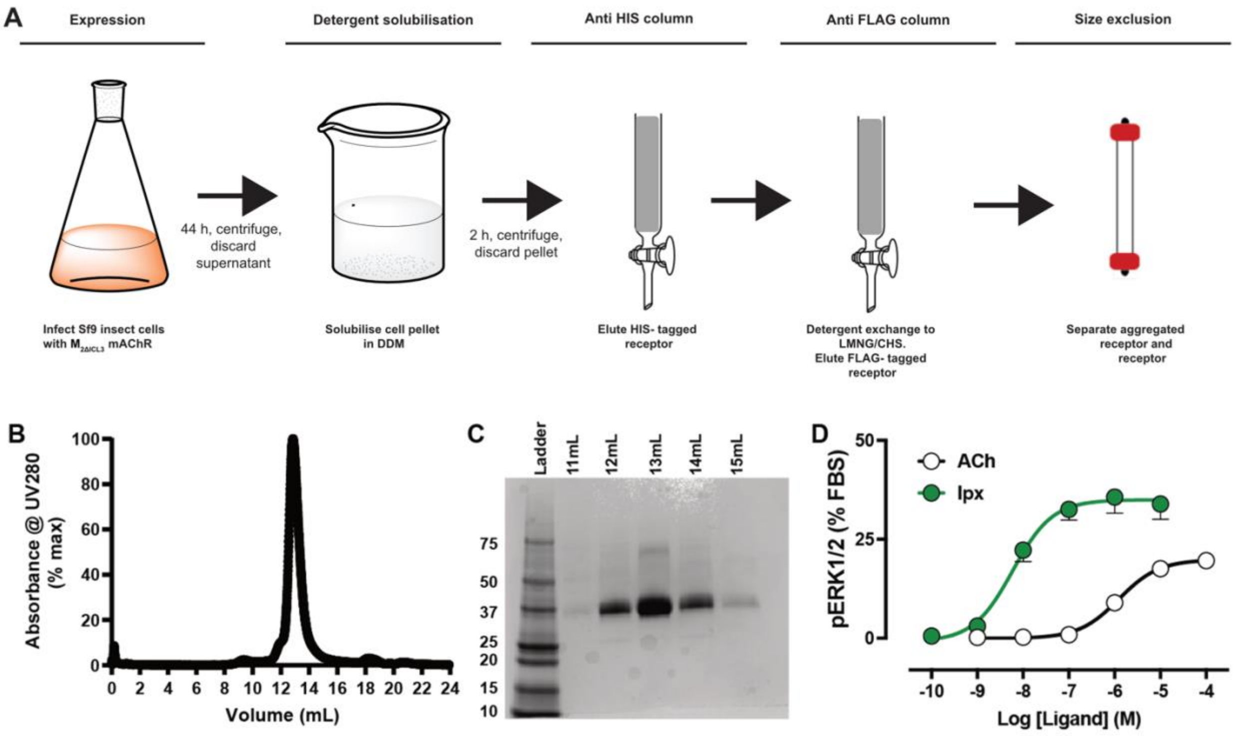

Fig. S1. Expression and purification of $M_{2\Delta ICL3}$ mAChR. (A) Expression and purification scheme of $M_{2\Delta ICL3}$ mAChR **(B)**. SEC trace of purified $M_{2\Delta ICL3}$ mAChR. **(C)** Coomassie staining of fractions obtained in **B**. **(D)** pERK phosphorylation concentration response curves for ACh and Ipx at the $M_{2\Delta ICL3}$ mAChR expressed in FlpIn CHO cells. Data are normalized to FBS response and represent the mean ± s.e.m. of seven independent experiments performed in duplicate. A three-parameter logistic equation was globally fit to the data to obtain pEC_{50} values of ACh: 5.91 ± 0.09 and Ipx: 8.23 ± 0.16.

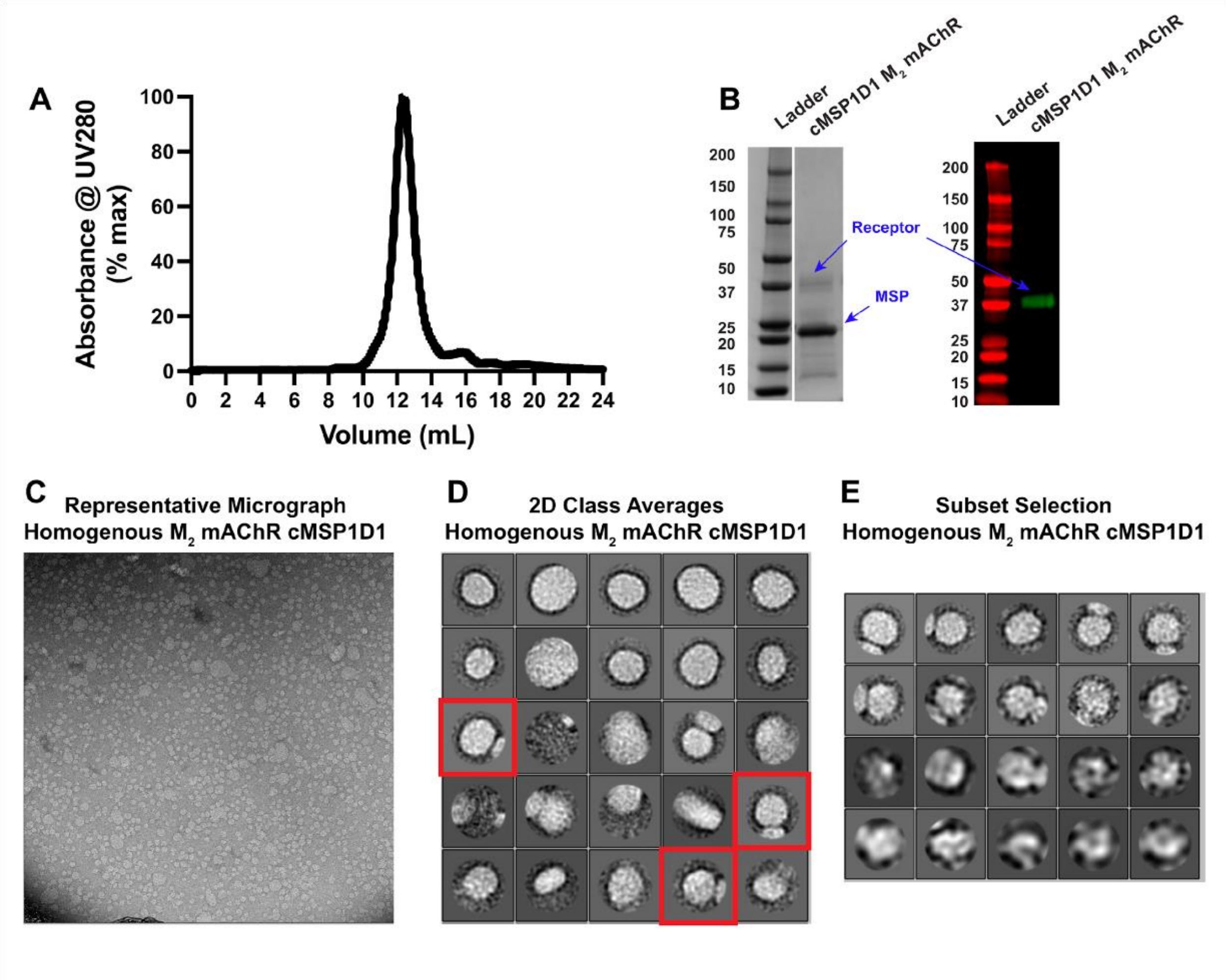

Fig. S2. Reconstitution of M_2 mAChR into nanodiscs. A) SEC traces following M_2 mAChR reconstitution into cMSP1D1 nanodiscs. **B)** The presence of receptor and MSP in each nanodisc was verified through left, SDS-Page with Coomassie staining and right, western blotting with anti-Flag antibody. **C)** Representative negative staining micrograph of M_2 mAChR nanodiscs complexed with anti-Flag FAB **D)** 2D class averages for the M_2 mAChR+Fab cMSP1D1 sample. 2D classes surrounded by a red border were selected and used to perform further subset selection 2D classification in **E)**.

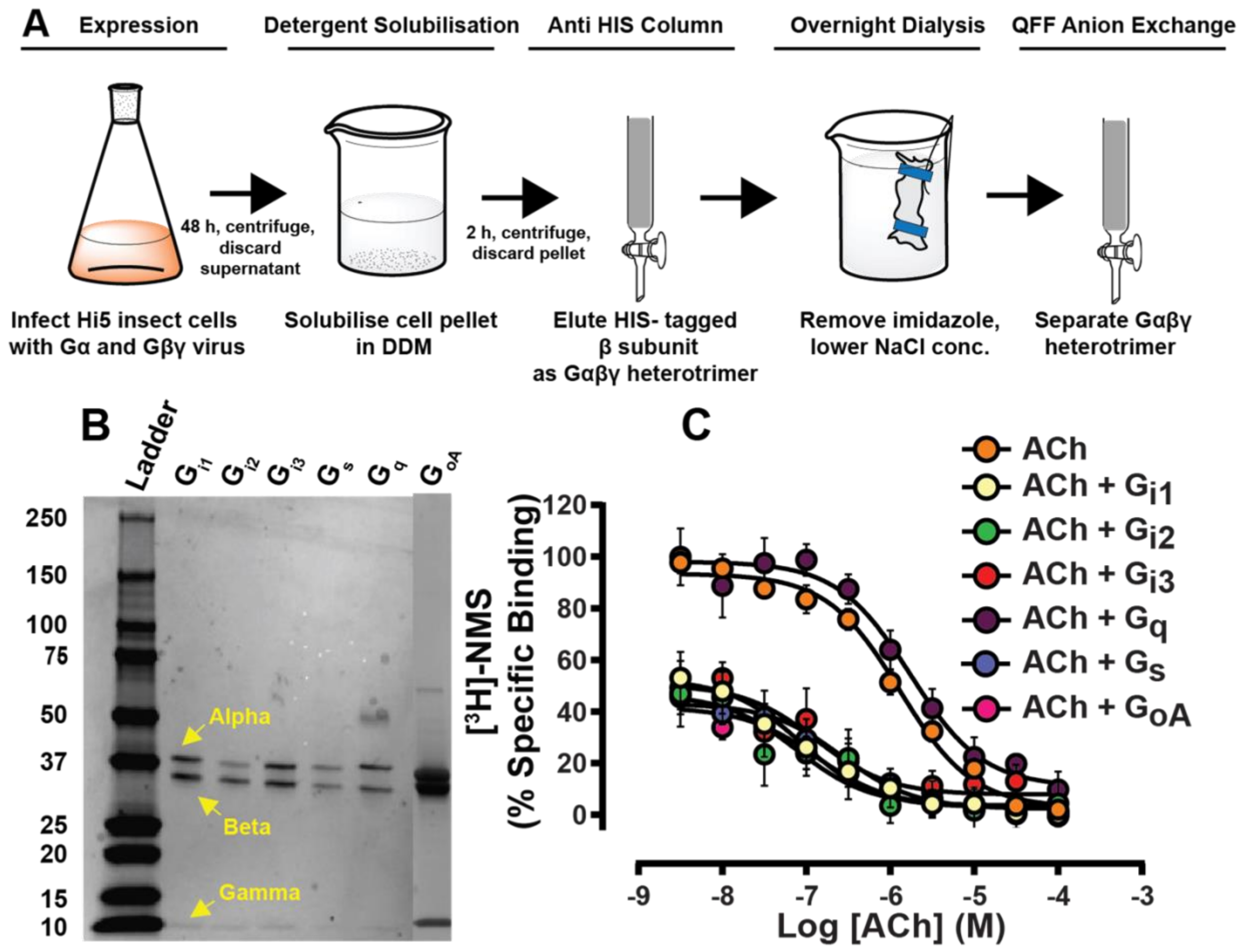

Fig. S3. Expression, purification and characterisation of WT G protein heterotrimers. (A) Expression and purification scheme for WT G protein heterotrimers. **(B)** Coomassie staining of purified WT G protein heterotrimers. **(C)** Competition binding of ACh and [^{3}H]-NMS in the presence of a saturating amount of G protein at M_2 mAChR cMSP1D1 nanodiscs. Data represent the mean ± s.e.m. of at least three independent experiments performed in duplicate.

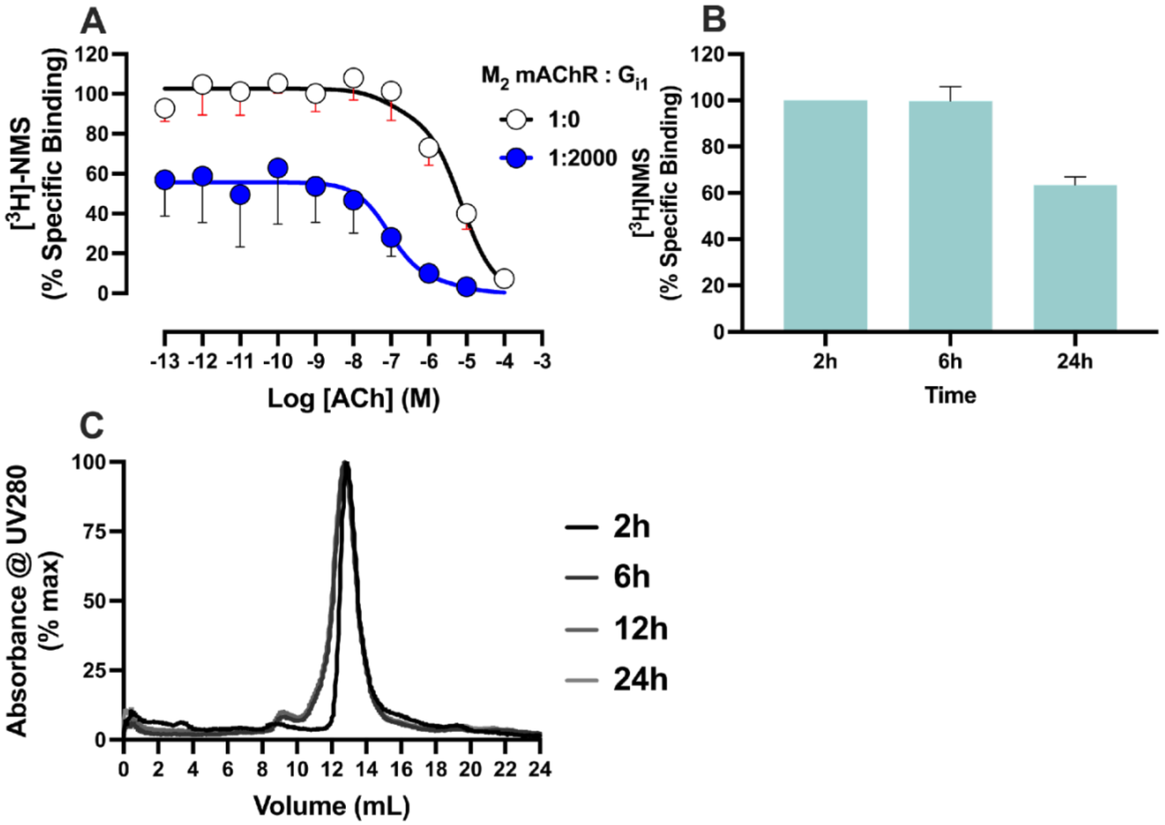

Fig. S4. Stability of M_2 mAChR nanodiscs. (A) Radioligand competition binding following 4 hours at room temperature between ACh and [^{3}H]-NMS at M_2 mAChR nanodiscs without and with a saturating amount G_{i1} protein. Data were normalised to the buffer only condition and represent the mean ± S.E.M of three individual experiments performed in duplicate. A two-state model of competition binding was fit to the data with p$K_{i\ (Low)}$ and p$K_{i\ (High)}$ values were shared. p$K_{i\ (Low)}$: 5.44 ± 0.31 and p$K_{i\ (High)}$: 7.17 ± 0.57 values for ACh were obtained. **(B)** Percentage of binding of a K_D concentration of [^{3}H]-NMS over time at M_2 mAChR nanodiscs. Data is normalized to the level of [^{3}H]-NMS binding observed at 2 hours. **(C)** SEC traces M_2 mAChR nanodiscs following incubations of different time periods at room temperature.

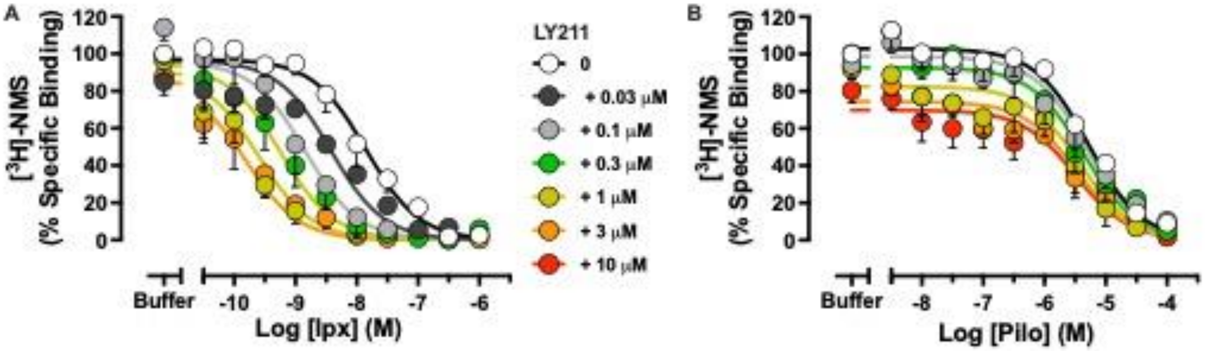

Fig. S5. Interaction binding of iperoxo and pilocarpine with LY2119620. Radioligand competition binding between **(A)** Ipx or **(B)** Pilo and [^{3}H]-NMS with increasing amounts of concentrations of LY211 at M_2 mAChR nanodiscs. For all panels, data represent the mean ± S.E.M. of at least three individual experiments performed in duplicate. Data were normalised to the buffer-only condition, and an allosteric ternary complex model was globally fit to the data. Group sizes and obtained parameters are listed in Table 1.

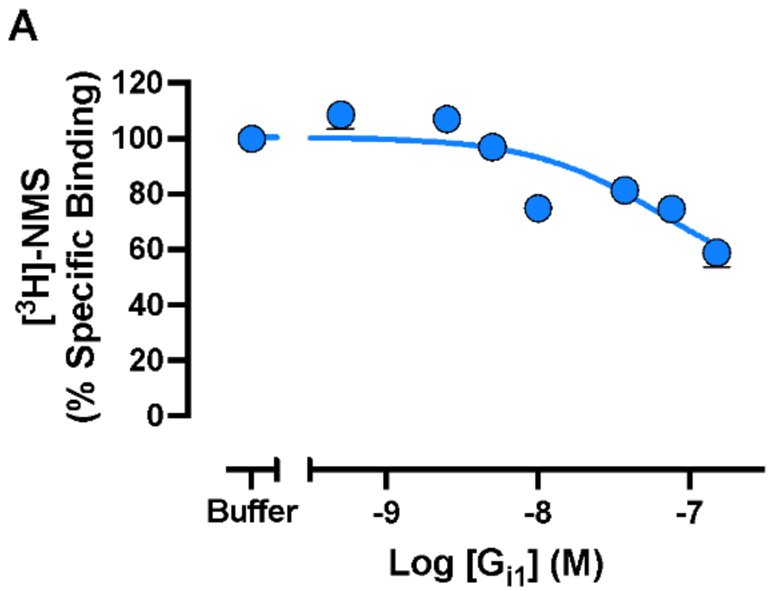

Fig. S6. G_{i1} heterotrimer protein versus [^{3}H]-NMS. Binding of [^{3}H]-NMS at M_2 mAChR nanodiscs in the presence of increasing concentrations G_{i1} heterotrimer. Data is replotted from buffer-only condition of Figure **2A**, **4A** and **4B** with G protein ratios converted to molar concentrations. Data were normalised to the buffer-only condition, followed by an allosteric ternary complex model being used to globally fit the data. Group sizes and obtained parameters are listed in Table 1.

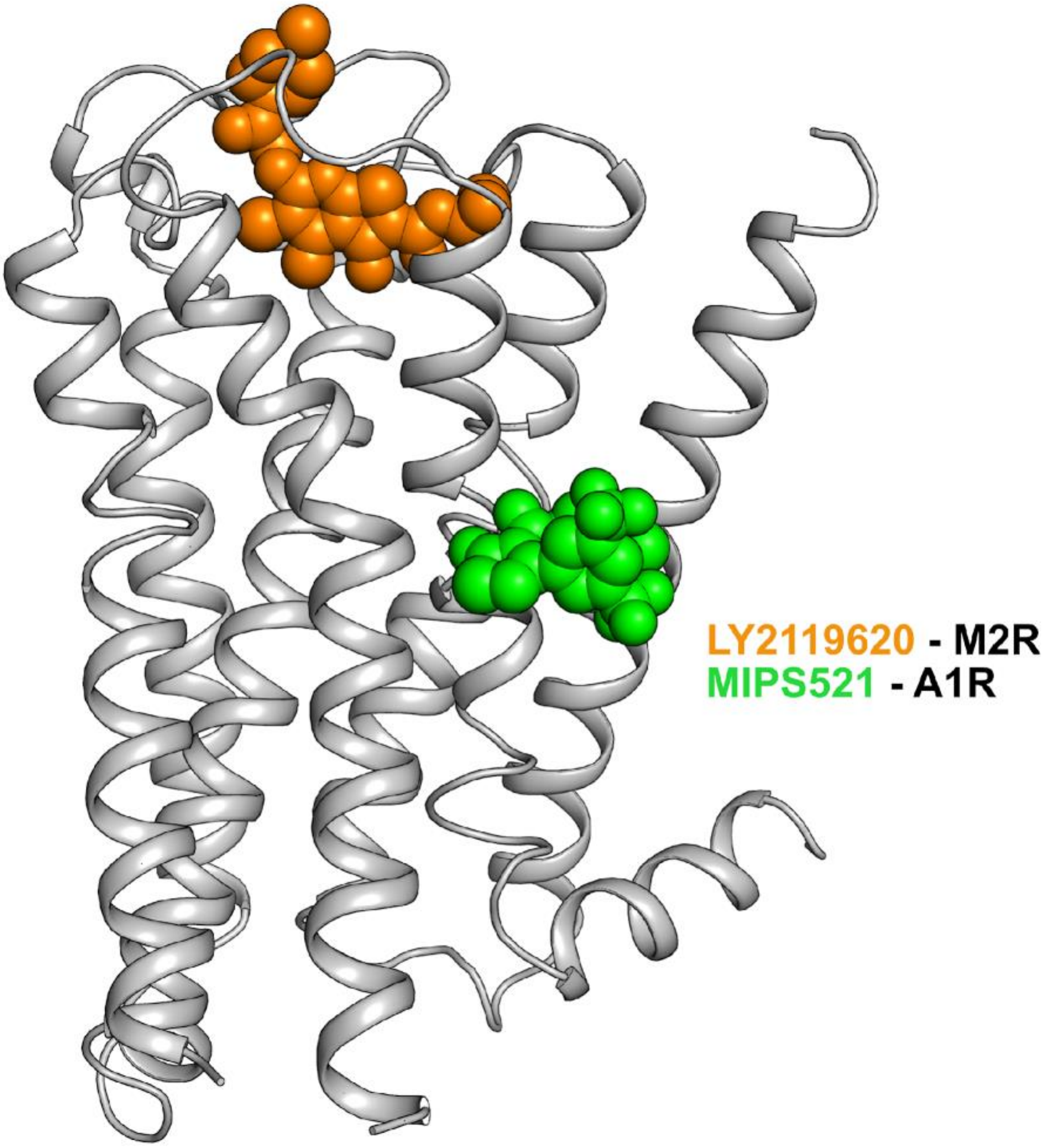

Fig. S7. Location of LY2119620 and MIPS521 binding sites. Comparison of the LY2119620 binding loci at the M_2 mAChR (PDB: 4MQT) and the MIPS521 binding loci at the A1AR (PDB: 7LD3).
